# Crescendo Angina Secondary to Congenital Absence of the Left Circumflex Coronary Artery: A Case Report

**DOI:** 10.7759/cureus.23749

**Published:** 2022-04-01

**Authors:** Esiemoghie J Akhigbe, Ebubechukwu Ezeh, Mohammad Amro, Olusola Olubowale, Gudjon Karlsson

**Affiliations:** 1 Internal Medicine, Marshall University Joan C. Edwards School of Medicine, Huntington, USA; 2 Internal Medicine, Misr University for Science and Technology, Cairo, EGY; 3 Cardiology, Marshall University Joan C. Edwards School of Medicine, Huntington, USA; 4 Cardiovascular Medicine, St. Mary’s Medical Center, Huntington, USA

**Keywords:** left circumflex artery (lcx), exertional chest pain, chest pain, refractory angina, congenital anomalies of coronary arteries

## Abstract

Congenital coronary artery abnormalities (CAAs) are very rare in the general population. Among these congenital anomalies, left circumflex artery (LCx) anomaly is the most common. Although many are asymptomatic, a small percentage of patients with this anomaly present with angina-like symptoms. Usually, a majority of these cases are found incidentally during coronary angiography. We present a 71-year-old male with crescendo angina with a positive chemical stress test. Coronary angiography showed an absent LCx and a superdominant right coronary artery (RCA). Although congenital LCx absence is a benign finding, the coexistence of this abnormality with significant atherosclerotic disease in the coronary artery can lead to significant morbidity and mortality in this population. Understanding the embryological and morphological significance of these anomalies is important in adequately diagnosing and managing these patients.

## Introduction

In general, coronary artery abnormalities (CAAs) can be defined as any coronary pattern or feature that is encountered in less than 1% of the general population [1]. Although most clinical manifestations are benign and asymptomatic, about 20% of cases manifest significant clinical symptoms [2]. ﻿Of all the coronary artery abnormalities, congenital absence of the left circumflex artery (LCx) is one of the most uncommon abnormalities with an incidence of 0.0067% [3]. Embryologically, this usually results from ﻿failure of the left circumflex artery to develop from the left atrioventricular groove [1].

Although congenital coronary artery anomalies are relatively uncommon, they are the second most common cause of sudden cardiac death (SCD) among young athletes. The risk of SCD in middle-aged or elderly individuals with an incidentally discovered coronary anomaly remains unclear [1]. The rarity and potential risk for complications make diagnosing this anomaly clinically relevant.

We present a case of congenital absence of the left circumflex artery found incidentally in an elderly male following presentation with angina-like symptoms.

## Case presentation

We present the case of a 71-year-old male with a past medical history significant for hypertension, diabetes, angina with coronary artery disease with previous left heart catheterization showing branch vessel disease, diagonal and apical left anterior descending artery (LAD) stenosis, abnormal stress test with EF of 33% with inferior basal wall ischemia, and a recent COVID-19 infection few months prior to presentation.

He presented to the emergency department with worsening chest pain, which was insidious and progressively worsening. He describes it as retrosternal with a dull/pressure-like sensation similar to previous chest pain but with increased intensity. He denied any orthopnea, paroxysmal nocturnal dyspnea (PND), palpitations, or syncope.

**Figure 1:**
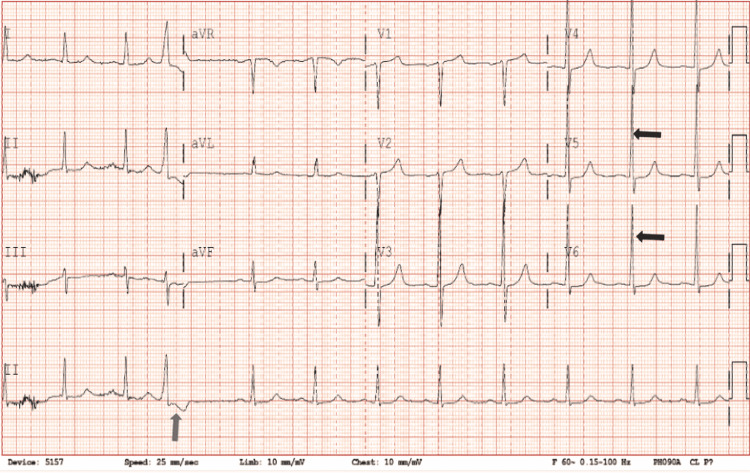
Electrocardiogram showing premature ventricular contractions (blue arrow) and possible left ventricular hypertrophy (black arrows).

His vitals showed a blood pressure of 128/80 mmHg with a pulse rate of 65 bpm, respiratory rate of 14, and SpO2 of 99% on room air. Physical examination was within normal limits, and the patient had no active chest pain. EKG performed showed a prolonged QT interval of 493, left ventricular hypertrophy, and multiple premature ventricular contractions (PVCs) (Figure 1).

**Figure 2:**
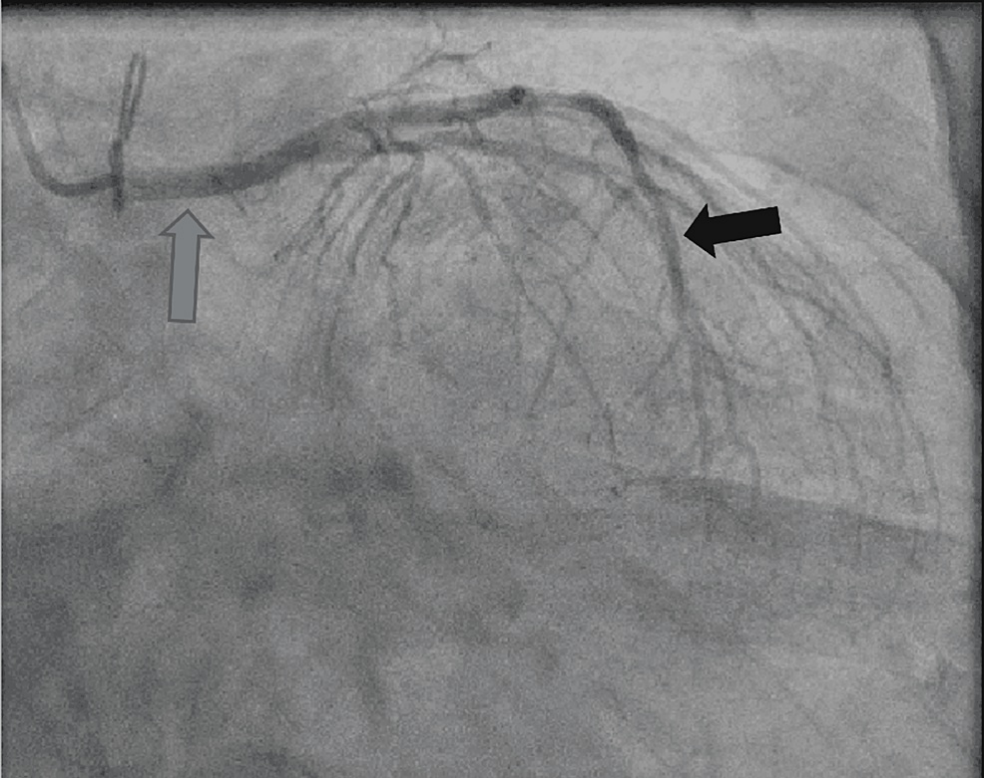
Cardiac catheterization showing absent left circumflex artery with extensive arterial disease. Blue arrow: left main artery; black arrow: left anterior descending artery

The patient then had a left heart catheterization that showed diffuse global hypokinesis, with abnormal coronary anatomy and the left main artery originating from the left coronary sinus in its usual fashion extending distally into the left anterior descending artery (LAD) with congenital absence of the left circumflex artery. There was a 40%-50% proximal LAD stenosis and an 80% mid-LAD stenosis (Figure 2).

**Figure 3:**
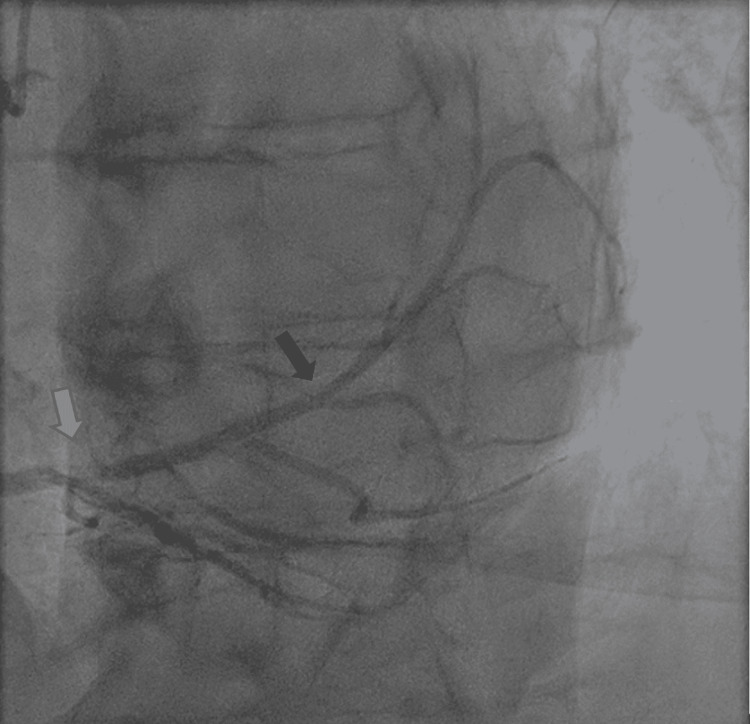
Right coronary artery showing >90% occlusion (blue arrow), and a large right posterior lateral branch extending and supplying a large surface area of the lateral wall (black arrow).

The right coronary artery (RCA) originated from the right coronary sinus of Valsalva and then courses its way to the lateral atrioventricular groove, giving rise to a large acute marginal branch and continues distally to supply the right posterior descending artery and a right large posterior lateral (PL) branch that extends to cover a very big area of the lateral wall that probably replaced the left circumflex artery territory. There was also a distal RCA stenosis of about 99% (Figure 3).

The patient had successful percutaneous transluminal coronary angioplasty (PTCA) and percutaneous coronary intervention (PCI) to the distal RCA/ostial PL branch, and PTCA and PCI to the mid-LAD. The patient was continued on optimized guideline-directed therapy and was referred to outpatient cardiac rehabilitation.

## Discussion

According to the literature, coronary artery abnormalities (CAAs) affect around 1% of the general population, ranging from 0.3% to 5.6% in studies on patients undergoing coronary angiography and in approximately 1% of routine autopsies [1].

In a study of 126,595 patients who underwent coronary arteriography during a 28-year interval, Yamanaka and Hubbs identified 1,686 consecutive patients with isolated congenital coronary artery anomalies (1.3% incidence). Of the patients found to have congenital artery abnormalities, just four patients (0.003%) had a congenital absence of the left circumflex artery [4]. In this condition, the lateral and posterior aspects of the left ventricle are supplied by a superdominant right coronary artery, a large diagonal artery, and a long right coronary artery continuing along the atrioventricular groove [5]. Superdominant RCA must exist in the case of an absent left circumflex artery. If a super dominant RCA does not exist, it must be evaluated for coronary artery hyperplasia syndrome (associated with hypoperfusion and dilated cardiomyopathy) and coronary origin anomaly (Bland-White-Garland syndrome) [6]. This phenomenon was proved by this patient who, on coronary angiography, showed a large right PL branch extending and supplying a large surface area of the lateral wall.

Although this anomaly is considered a benign condition in the absence of coronary occlusive disease, it can cause angina-like symptoms, particularly on exertion. It has been hypothesized that the “steal phenomenon” could explain symptoms, where increased metabolic demand in the LCx territory would cause ischemia in the LAD or RCA territories [1]. In addition to the absent LCx, some concomitant congenital abnormalities have been described in the literature, including an atretic mid-LAD originating from the sinus of Valsalva, dual LAD, a LAD originating from the right coronary cusps, and a non-existing subclavian artery [7,8].

Diagnosis of absent LCx is confirmed by computed tomography (CT) coronary angiogram, which is highly accurate for the evaluation of congenital coronary artery anomalies because it can present a three-dimensional image and demonstrate the anatomical origin, course, and termination of coronary artery anomalies and define their relationship to other cardiac and noncardiac structures [2]. It can also help differentiate between a congenital absence, an anomalous LCx, or complete occlusion of the left circumflex artery. As a result of its benign nature, ﻿treatment is usually as per guideline-directed medical therapy, and no specific lesion intervention has been proposed.

This patient who presented with chest pain and progressively worsening angina with an absent LCx on coronary angiography causes a conundrum in the diagnosis. This patient with worsening atherosclerosis artery of the RCA is burdened with the increased metabolic responsibility of supplying the region of the absent left circumflex artery. This causes ischemia-like symptoms as seen on the patient’s stress test.

A better understanding of the embryology and natural history of this anomaly would make diagnosis less of a challenge.

## Conclusions

Congenital absence of the left circumflex artery is an extremely rare abnormality. Its presence is found incidentally upon investigation for angina-like symptoms. Its most common presentation is chest pain with some EKG findings. This pathology can present in isolation or with other coronary anomalies, thus making it clinically relevant. A definitive diagnosis should be done with a CT coronary angiogram to help minimize misdiagnosis.

## References

[1] Villa AD, Sammut E, Nair A, Rajani R, Bonamini R, Chiribiri A (2016). Coronary artery anomalies overview: the normal and the abnormal. World J Radiol.

[2] Hongsakul K, Suwannanon R (2012). Congenital absence of left circumflex artery detected by computed tomography coronary angiography: a case report. Case Rep Vasc Med.

[3] Fugar S, Issac L, Okoh AK, Chedrawy C, Hangouche NE, Yadav N (2017). Congenital absence of left circumflex artery: a case report and Rreview of the literature. Case Rep Cardiol.

[4] Yamanaka O, Hobbs RE (1990). Coronary artery anomalies in 126,595 patients undergoing coronary arteriography. Cathet Cardiovasc Diagn.

[5] Ali FS, Khan SA, Tai JM, Fatimi SH, Dhakam SH (2009). Congenital absence of left circumflex artery with a dominant right coronary artery. BMJ Case Rep.

[6] Bildirici U, Kılıç T, Ural D, Bulut O, Ural E (2011). Combined congenital coronary artery anomaly: dual left anterior descending coronary artery and absence of left circumflex artery. Anadolu Kardiyol Derg.

[7] Teunissen PF, Marcu CB, Knaapen P, van Royen N (2015). A case of multiple coronary atresias: a rarity even within the family of coronary anomalies. Eur Heart J.

[8] Harada R, Nakajima T, Kawaharda N (2017). Absent left circumflex artery detected by computed tomography-angiography. Asian Cardiovasc Thorac Ann.

